# Early Infection Incidence and Risk of Acute Leukemia Development Among Mexican Children

**DOI:** 10.3390/cancers17050733

**Published:** 2025-02-21

**Authors:** Omar Sepúlveda-Robles, Janet Flores-Lujano, Juan Carlos Núñez-Enríquez, Elva Jiménez-Hernández, David Aldebarán Duarte-Rodríguez, Jorge Alfonso Martín-Trejo, Laura Eugenia Espinoza-Hernández, Xochiketzalli García-Jiménez, Rogelio Paredes-Aguilera, Juan José Dosta-Herrera, Javier Anastacio Mondragón-García, Heriberto Valdés-Guzmán, Laura Mejía-Pérez, Gilberto Espinoza-Anrubio, María Minerva Paz-Bribiesca, Perla Salcedo-Lozada, Rodolfo Ángel Landa-García, Rosario Ramírez-Colorado, Luis Hernández-Mora, Marlene Santamaría-Ascencio, Anselmo López-Loyola, Arturo Hermilo Godoy-Esquivel, Luis Ramiro García-López, Alison Ireri Anguiano-Ávalos, Karina Mora-Rico, Alejandro Castañeda-Echevarría, Roberto Rodríguez-Jiménez, José Alberto Cibrian-Cruz, Rocío Cárdenas-Cardos, Martha Beatriz Altamirano-García, Martin Sánchez-Ruiz, Roberto Rivera-Luna, Luis Rodolfo Rodríguez-Villalobos, Francisco Hernández-Pérez, Jaime Ángel Olvera-Durán, Luis Rey García-Cortés, José Refugio Torres-Nava, Marlon De Ita, Aurora Medina-Sanson, Minerva Mata-Rocha, José Gabriel Peñaloza-Gonzalez, Rosa Martha Espinosa-Elizondo, Luz Victoria Flores-Villegas, Raquel Amador-Sanchez, Darío Orozco-Ruiz, Maria Luisa Pérez-Saldívar, Martha Margarita Velázquez-Aviña, Laura Elizabeth Merino-Pasaye, Karina Anastacia Solís-Labastida, Ana Itamar González-Ávila, Jessica Denisse Santillán-Juárez, Vilma Carolina Bekker-Méndez, Silvia Jiménez-Morales, Angélica Rangel-López, José Arellano-Galindo, Jorge Meléndez-Zajgla, Haydeé Rosas-Vargas, Juan Manuel Mejía-Aranguré

**Affiliations:** 1 Unidad de Investigación Médica en Genética Humana, UMAE Hospital de Pediatría, Centro Médico Nacional “Siglo XXI”, Instituto Mexicano del Seguro Social (IMSS), Mexico City 06720, Mexico; sero__82@hotmail.com (O.S.-R.); morgan_lee77@hotmail.com (M.D.I.); mmata@conacyt.mx (M.M.-R.); 2 Unidad de Investigación Médica en Epidemiología Clínica, UMAE Hospital de Pediatría, Centro Médico Nacional “Siglo XXI”, Instituto Mexicano del Seguro Social (IMSS), Mexico City 06720, Mexico; janetflores22@yahoo.com.mx (J.F.-L.); jcarlos_nu@hotmail.com (J.C.N.-E.); turunci@gmail.com (D.A.D.-R.); maria_luisa_2000_mx@yahoo.com (M.L.P.-S.); 3 Servicio de Hematología Pediátrica, Hospital General “Gaudencio González Garza”, Centro Médico Nacional “La Raza”, Instituto Mexicano del Seguro Social (IMSS), Mexico City 02990, Mexico; elvajimenez@yahoo.com (E.J.-H.); laura.espinoza@imss.gob.mx (L.E.E.-H.); har_roam@hotmail.com (X.G.-J.); 4 Servicio de Hematología Pediátrica, UMAE Hospital de Pediatría, Centro Médico Nacional “Siglo XXI”, Instituto Mexicano del Seguro Social (IMSS), Mexico City 06720, Mexico; jorge.martin.trejo@gmail.com (J.A.M.-T.); kas_anastacia@yahoo.com (K.A.S.-L.); 5 Servicio de Hematología, Instituto Nacional de Pediatría (INP), Secretaría de Salud (SS), Mexico City 04530, Mexico; rapa3852@yahoo.com; 6 Servicio de Cirugía Pediátrica, Hospital General “Gaudencio González Garza”, Centro Médico Nacional “La Raza”, Instituto Mexicano del Seguro Social (IMSS), Mexico City 02990, Mexico; juan.dosta@imss.gob.mx; 7 Servicio de Cirugía Pediátrica, HGR No. 1 “Dr. Carlos Mac Gregor Sánchez Navarro”, Instituto Mexicano del Seguro Social (IMSS), Mexico City 03103, Mexico; drjaiercirped@gmail.com; 8 Hospital Pediátrico de Iztacalco, Secretaría de Salud de la Ciudad de México (SSCDMX), Mexico City 08310, Mexico; heribertovaldesg@yahoo.com; 9 Hospital Pediátrico de Iztapalapa, Secretaría de Salud de la Ciudad de México (SSCDMX), Mexico City 09070, Mexico; laurimepe_3@hotmail.com; 10 Servicio de Pediatría, Hospital General Zona (HGZ) No. 8 “Dr. Gilberto Flores Izquierdo”, Instituto Mexicano del Seguro Social (IMSS), Mexico City 01090, Mexico; gilberto.espinozaa@yahoo.com.mx (G.E.-A.); martha.altamirano@imss.gob.mx (M.B.A.-G.); 11 Servicio de Pediatría, Hospital Juárez del Centro, Secretaría de Salud (SS), Mexico City 06090, Mexico; qfbmine@hotmail.com; 12 Hospital General de Ecatepec “Las Américas”, Instituto de Salud del Estado de México (ISEM), Ecatepec 55076, Mexico; salcedo_perla@yahoo.com.mx (P.S.-L.); masaruiz@yahoo.com (M.S.-R.); 13 Hospital General “Dr. Manuel Gea González” Secretaría de Salud (SS), Mexico City 14080, Mexico; rodolfoangel.landa@gmail.com; 14 Hospital Pediátrico “La Villa”, Secretaría de Salud de la Ciudad de México (SSCDMX), Mexico City 07050, Mexico; rosarioram@yahoo.com.mx; 15 Hospital Pediátrico “San Juan de Aragón”, Secretaría de Salud de la Ciudad de México (SSCDMX), Mexico City 07969, Mexico; luismhm64@hotmail.com; 16 Servicio de Pediatría, HGR No. 72 “Lic. Vicente Santos Guajardo”, Instituto Mexicano del Seguro Social (IMSS), Mexico City 54030, Mexico; marlene.santamaria@imss.gob.mx; 17 Servicio de Cirugía Pediátrica, HGZ No. 32, Instituto Mexicano del Seguro Social (IMSS), Mexico City 04980, Mexico; anselmo.lopez@imss.gob.mx; 18 Servicio de Cirugía Pediátrica, Hospital Pediátrico de Moctezuma, Secretaría de Salud de la Ciudad de México (SSCDMX), Mexico City 15530, Mexico; poncho9001@yahoo.com.mx; 19 Servicio de Pediatría, Hospital Pediátrico de Tacubaya, Secretaría de Salud de la Ciudad de México (SSCDMX), Mexico City 11870, Mexico; dr.ragalo@gmail.com (L.R.G.-L.); rodolforguezv@yahoo.com.mx (L.R.R.-V.); 20 Urgencias Pediátricas, HGZ No. 47, Instituto Mexicano del Seguro Social (IMSS), Mexico City 09200, Mexico; alisonireri@yahoo.com.mx (A.I.A.-Á.); turco168@hotmail.com (F.H.-P.); 21 Servicio de Cirugía Pediátrica, Hospital Regional “1° Octubre”, Instituto de Seguridad Social al Servicio de los Trabajadores del Estado (ISSSTE), Mexico City 07760, Mexico; morazul26@yahoo.com (K.M.-R.); cpjaod@yahoo.com.mx (J.Á.O.-D.); 22 Servicio de Pediatría, HGR No. 25, Instituto Mexicano del Seguro Social (IMSS), Mexico City 09208, Mexico; alejandro.echevarria@imss.gob.mx; 23 Servicio de Pediatría, Hospital General de Zona con Medicina Familiar (HGZMF) No. 29, Instituto Mexicano del Seguro Social (IMSS), Mexico City 07950, Mexico; roberto.rodriguez@imss.gob.mx; 24 Servicio de Cirugía Pediátrica, HGZ No. 27, Instituto Mexicano del Seguro Social (IMSS), Mexico City 06900, Mexico; cibriandrped@yahoo.com; 25 Servicio de Oncología, Instituto Nacional de Pediatría (INP), Secretaría de Salud (SS), Mexico City 04530, Mexico; oncoped_inp@hotmail.com (R.C.-C.); riveraluna@terra.com.mx (R.R.-L.); 26 Delegación Regional Estado de México Oriente, Instituto Mexicano del Seguro Social (IMSS), Mexico City 54060, Mexico; luis.garciaco@imss.gob.mx; 27 Servicio de Oncología, Hospital Pediátrico de Moctezuma, Secretaría de Salud de la Ciudad de México (SSCDMX), Mexico City 15530, Mexico; torresoncoped@live.com.mx (J.R.T.-N.); dario.or@hotmail.com (D.O.-R.); 28 Departamento de Genética y Biología Molecular, Centro de Investigaciones y de Estudios Avanzados del Instituto Politécnico Nacional (CINVESTAV-IPN), Mexico City 07360, Mexico; 29 Servicio de Hemato-Oncología, Hospital Infantil de México Federico Gómez, Secretaría de Salud (SS), Mexico City 06720, Mexico; auroramedina@aol.com.mx; 30 Servicio de Onco-Pediatría, Hospital Juárez de México, Secretaría de Salud (SS), Mexico City 07760, Mexico; penaloza_6@yahoo.es (J.G.P.-G.); m_mvelazquez@yahoo.com.mx (M.M.V.-A.); 31 Servicio de Hematología Pediátrica, Hospital General de México, Secretaría de Salud (SS), Mexico City 06720, Mexico; rmespinosa1605@hotmail.com; 32 Servicio de Hematología Pediátrica, Centro Médico Nacional “20 de Noviembre”, Instituto de Seguridad Social al Servicio de los Trabajadores del Estado (ISSSTE), Mexico City 03104, Mexico; victoriabanco@yahoo.com.mx (L.V.F.-V.); sketch0712@gmail.com (L.E.M.-P.); 33 Hospital General Regional No. 1 “Carlos McGregor Sánchez Navarro”, Instituto Mexicano del Seguro Social (IMSS), Mexico City 03100, Mexico; dibs_amador@hotmail.com (R.A.-S.); itamarga@hotmail.com (A.I.G.-Á.); 34 Servicio de Hemato-Oncología Pediátrica, Hospital Regional 1° de Octubre, Instituto de Seguridad Social al Servicio de los Trabajadores del Estado (ISSSTE), Mexico City 07760, Mexico; jessydenise22@hotmail.com; 35 Unidad de Investigación Médica en Inmunología e Infectología, Hospital de Infectología “Dr. Daniel Méndez Hernández”, Centro Médico Nacional “La Raza”, Instituto Mexicano del Seguro Social (IMSS), Mexico City 02990, Mexico; bekkermendez@yahoo.com; 36 Laboratorio de Medicina de Precisión, Instituto Nacional de Medicina Genómica (INMEGEN), Mexico City 14610, Mexico; sjimenez@inmegen.gob.mx; 37 Coordinación de Investigación en Salud, Instituto Mexicano del Seguro Social (IMSS), Mexico City 06720, Mexico; ragn62@prodigy.net.mx; 38 Laboratorio de Virología, Unidad de Investigación en Enfermedades Infecciosas, Hospital Infantil de México “Dr. Federico Gómez”, Secretaría de Salud (SS), Mexico City 06720, Mexico; josearellanogalindo@yahoo.com.mx; 39 Laboratorio de Genómica Funcional del Cáncer, Instituto Nacional de Medicina Genómica (INMEGEN), Mexico City 14610, Mexico; jmelendez@inmegen.gob.mx; 40 Facultad de Medicina, Universidad Nacional Autónoma de México (UNAM), Mexico City 04360, Mexico

**Keywords:** acute leukemia, Latino population, maternal infections, infection incidence

## Abstract

Background: The few epidemiologic studies of infection exposure in early life and acute leukemia (AL) risk in Latino children have yielded inconsistent results, suggesting a possible effect of ethnicity. Here, we examined the correlation between infection exposure and acute leukemia risk in children from Mexico City—One of the biggest Latino cities worldwide. Methods: This study included 1455 Mexican children diagnosed with de novo AL (2002–2016), and 1455 control individuals frequency-matched by age and health institution. The AL population included acute lymphoblastic leukemia (ALL), Pre-B ALL, and acute myeloblastic leukemia (AML). Logistic regression analyses were performed to investigate direct and indirect proxies of infection in children or their mothers. Results: Upper respiratory tract infections during the child’s first year of life were a risk factor for AL (OR, 2.76; 95% CI, 1.48–5.15), including ALL (OR, 3.14; 95% CI, 1.67–5.89) and Pre-B (OR, 3.11; 95% CI, 1.63–5.96). Mother’s infections before and during pregnancy were protective factors against AL (OR, 0.55; 95% CI, 0.47–0.64; and OR, 0.61; 95% CI, 0.52–0.72, respectively). These associations included ALL and Pre-B. In contrast, only mothers’ infections before pregnancy and respiratory tract infections were protective factors against AML (OR, 0.45; 95% CI, 0.33–0.62; and OR, 0.50; 95% CI, 0.37–0.68, respectively). Conclusions: Infections during the first year of life were associated with AL development in children of Mexico City. Additionally, mothers’ exposure to respiratory tract infections before and during pregnancy reduced the AL risk in this Latino population.

## Simple Summary

The majority of studies in non-Latino populations show that exposure to infections in early life reduces the risk of acute leukemia development; however, studies among Latino children have yielded inconsistent results, suggesting differences between ethnic groups. The aim of this study was to examine the correlation between exposure to infections and childhood leukemia among Latino children from Mexico City—A population with one of the highest incidence rates of acute leukemia worldwide. The present findings showed that upper respiratory tract infections during a child’s first year of life were associated with acute leukemia development. Additionally, the mothers’ exposure to respiratory tract infections before or during pregnancy emerged as an important factor associated with reduced AL risk in this Latino-children population. Moreover, the present results indicated a probable extension of the window effect during which infection incidence can influence AL risk, but further studies are needed.

## 1. Introduction

Acute leukemia (AL) is the most common childhood cancer worldwide, accounting for one-third of all cancer cases among children under 15 years old [1]. Both acute myeloblastic leukemia (AML) and acute lymphocytic leukemia (ALL) occur in children, but ALL accounts for 80% of all childhood AL cases [2]. The incidence of ALL varies among ethnic groups, with the Latin American population showing higher incidence rates and poorer outcomes [2]. A recent study reports that Mexico City has an age-standardized AL incidence rate of 63.3 cases per million, which is the highest incidence rate reported worldwide [3].

There is a need to study the genetic and environmental factors affecting the etiology of ALL, and how these could impact its incidence rates. Genetic factors associated with a higher risk of ALL development include Bloom syndrome, neurofibromatosis, Fanconi anemia, ataxia telangiectasia, and trisomy 21 [4]. However, these conditions are present in less than 1% of all cases [4], suggesting the greater importance of external factors, such as exposure to tobacco smoke, pesticides, hydrocarbons, or ionizing radiation [5,6,7,8]. Factors related to early stimulation of the immune system, such as infections, have long been proposed to play a role in childhood leukemia etiology [9,10,11,12]. According to Mel Greaves’ hypothesis, ALL evolves from a prenatal mutation that generates a preleukemic clone, and a subsequent abnormal immune response to a common infection triggers secondary changes that drive conversion to overt leukemia [13]. In this context, exposure to infections in early life is thought to reduce the risk of an abnormal immune response to common infection, by preventing future abnormal responses and thereby influencing the fate of pre-leukemic clones [13].

Although no specific infectious agent has been identified, epidemiological studies have assessed direct (e.g., infection history) and indirect (e.g., birth order, daycare attendance, and breastfeeding) measures of early immune stimulation, and the results suggest that exposure to infections has some impact on leukemogenesis [14,15,16,17,18,19,20,21,22,23,24,25]. Among such studies conducted in non-Latino populations, the majority support Greaves’ hypothesis that early infections are protective against ALL development; however, studies among Latino children have yielded diverse results, suggesting ethnicity-related differences [19,20,22,25]. In a study of Latino children residing in California, USA, Ma et al. (2005) reported that AL was not associated with daycare attendance, breastfeeding, or infections in early life [22]. Urayama et al. (2011) found that daycare attendance by 6 months of age and birth order were associated with reduced ALL risk among non-Latino children but not among Latino children, whereas ear infection before 6 months was protective in both ethnic groups, supporting Greaves’ hypothesis [25]. In a study of children in Costa Rica, Figueroa et al. (2020) showed that contact with any pet or any farm animal (surrogate proxies of infection) was associated with a reduced risk of ALL, whereas experiencing a fever lasting longer than one week (a putative proxy of severe infection) was associated with increased ALL risk [19]. These varying results between studies could be related to the imprecision of epidemiologic instruments for adequately measuring early-life infections or the small population sample sizes, rather than due to a true ethnicity-based difference in ALL etiology [25].

We previously assessed breastfeeding and early infection in the etiology of childhood AL among Mexican children with Down Syndrome. We found that breastfeeding (≤6 months) was a protective factor against ALL development, while hospitalization due to infection during the first year of life increased ALL risk, which does not support Greaves’ hypothesis of early infection being a protective factor against AL development [20]. However, it is unknown whether these results are applicable to children with ALL and without Down Syndrome in Mexico City. Therefore, in the present study, we analyzed the impact of early infections on the pathogenesis of leukemia, by assessing birth order, breastfeeding, maternal infections during pregnancy, and early-life infections in children with AL in Mexico City. To our knowledge, this is the largest study of a Latino population performed by the Mexican Interinstitutional Group for the Identification of the Causes of Childhood Leukemia (MIGICCL).

## 2. Materials and Methods

### 2.1. Study, Cases, and Controls

The MIGICCL performed a case–control study including children under 18 years of age (1455 cases and 1455 controls), who were diagnosed with AL during the period 2010–2015. Patients were identified from 31 public hospitals of Mexico City and the metropolitan area, under the auspices of the Instituto Mexicano del Seguro Social (IMSS), Instituto de Seguridad Social al Servicio de los Trabajadores del Estado (ISSSTE), Secretaría de Salud (SS), and Secretaría de Salud del Distrito Federal (SSDF). All cases were diagnosed with acute leukemia (AL), including acute lymphoblastic leukemia (ALL) and acute myeloid leukemia (AML), based on the morphologic and immunophenotypic features of blasts. The controls were children without AL, who were frequency-matched with cases according to age and health institution. If no age-matched control was found for a case after three visits to the same hospital, a control was selected with an age within ±2 years of the case, regardless of sex. The control group excluded children with any neoplasms, hematological diseases, allergic diseases, acute infections, or congenital malformation/birth defects (visible or previously diagnosed). The controls’ diagnoses included surgical cases (59.7%), including tonsillectomy, orchidopexy, circumcision, hernioplasty, fractures, appendectomy, and other surgical diseases; and non-surgical diseases (40.3%), including gastroesophageal reflux disease, epilepsy, head trauma, intoxications, migraine, closed fractures, sprain, myopia, astigmatism, and burns. Written informed consent or assent (for children ≥8 years old) was obtained from parents before their children participated in this study. The study was approved by the Ethics and National Committee of Scientific Research, and by the ethics committees of all participating health institutions.

### 2.2. Data Collection

Clinical information required for this study was collected by staff using previously standardized questionnaires (QMOD website; National Cancer Institute, 1998). Parents who agreed to participate were individually interviewed about their child’s medical history and demographic data, including sex, age at diagnosis, birth order, birth weight, breastfeeding, infections during the first year of life (upper and lower respiratory tract infections, gastrointestinal infections, and others), infections requiring hospitalization during the first year of life (upper and lower respiratory tract infections, gastrointestinal infections, and others), mother’s infections during pregnancy (respiratory tract infections, gastrointestinal infections, urinary tract infections, vaginal infections, and others), family history of cancer, parents’ age at pregnancy, parents’ education levels, and sociodemographic aspects. As an indicator of socioeconomic status, we used stacking level, which was calculated according to the number of persons per room in the household, classified using the criteria of Bronfman et al. (1988) [26] as follows: not crowded (<1.5 persons per room), semi-crowded (1.6–3.5), and crowded (≥3.6). Both crowded and semi-crowded were considered to indicate a low socioeconomic level.

### 2.3. Statistical Analyses

Descriptive statistical analyses were performed, and frequency measurements and percentages were calculated. The odds ratios (ORs) and the 95% confidence intervals (CIs) were calculated for the relationships between breastfeeding, early infections, hospitalization due to infections, mother’s infections during pregnancy, and the control variables for AL, ALL, and AML. Independent variables (breastfeeding, early infections, hospitalization due to infections, and mother’s infections during pregnancy) were stratified for each control variable, to control for confounding factors. ORs were estimated using unconditional logistic regression analysis, with adjustment for the children’s data, such as sex, age, birth weight, birth order, and standard of living. A logistic regression model was also applied. Statistical analyses were performed using SPSS IBM (Statistical Package for the Social Sciences, Inc., Version 26.0, Chicago, IL, USA).

## 3. Results

This study included 1455 children (53.5% boys and 46.5% girls) diagnosed with AL (Table 1). AL was most commonly diagnosed at 5–15 years of age (53.4%), and the majority of diagnoses were ALL (86.1%), followed by AML (13.9%). The majority of parents had less than 9 years of education. Most parents were <35 years of age at conception of their child, with a median age of 24.9 years for mothers, and 27.1 years for fathers (Table 1).

Cases and controls significantly differed with regard to gender and age at diagnosis; however, these differences were not relevant from a numerical perspective due to the large sample size analyzed. Notably, the groups did not significantly differ in socioeconomic level or the mother’s education level (Table 1).

Descriptive analysis indicated that infections during a child’s first year of life were not associated with AL development. However, in the analysis stratified by infection type, upper respiratory tract infections were associated with an increased risk of AL (OR, 2.67; 95% CI, 1.44–4.96) (Table 1). Increased risk of AL was also associated with birth weight ≥ 3500 g, family history of cancer, father’s education level (≥13 years and ≤9 years), and breastfeeding (OR, 1.53; 95% CI, 1.20–1.95) (Table 1). On the other hand, reduced AL risk was associated with firstborn children (OR, 0.81; 95% CI, 0.69–0.93), and mother’s infections (especially respiratory infections) before pregnancy (OR, 0.54; 95% CI, 0.47–0.63) and during pregnancy (OR, 0.60; 95% CI, 0.51–0.69) (Table 1). AL development was not associated with hospitalization due to infection during the child’s first year of life, including in analyses stratified by type of infection (Table 1).

Due to the difficulties of matching cases and controls for age at diagnosis and sex, these two variables were included in the logistical regression analysis (Table 2 and Table 3). The results of this analysis revealed no association between infections during the child’s first year of life and AL. However, in the stratified analysis by type of infections, upper respiratory tract infections were identified as a risk factor for AL development (OR, 2.76; 95% CI, 1.48–5.15). This risk was increased among cases of ALL (OR, 3.14; 95% CI, 1.67–5.89) and of the Pre-B leukemia subtype (OR, 3.11; 95% CI, 1.63–5.96) (Table 2). In contrast, a reduced risk of AL (including ALL and Pre-B subtype leukemia) was associated with mother’s infections before pregnancy (OR, 0.55; 95% CI, 0.47–0.64) and during pregnancy (OR, 0.61; 95% CI, 0.52–0.72) (Table 3). Although both variables showed similar associations, only infections before pregnancy were associated with a reduced risk of AML development (OR, 0.50; 95% CI, 0.37–0.68). The stratified analysis showed that the association was related to respiratory tract infections (OR, 0.45; 95% CI, 0.33–0.62) (Table 3). Gastrointestinal and urinary tract infections were also associated with a reduced risk of AL (Table 3).

## 4. Discussion

In this population-based study, we analyzed the correlation between exposure to infections and leukemia in children from Mexico City—A Latin population with one of the highest AL incidence rates worldwide. Our results showed that infections during the child’s first year of life were not associated with AL. However, analysis stratified by infection types revealed that upper respiratory tract infections were associated with increased AL risk, specifically, ALL and Pre-B ALL. Interestingly, mothers’ infections before and during pregnancy were associated with reduced risks of ALL and Pre-B, while only infections before pregnancy and respiratory tract infections were associated with a reduced risk of AML.

In the context of Greaves’ hypothesis that early infection is protective against developing acute leukemia, our results elucidated two likely risk outputs, which depend on whether exposition exposure occurs directly (early life or postnatally) or indirectly in mothers (in utero or prenatally). For example, the risk of developing acute leukemia was higher among children who experienced upper respiratory tract infections during early life, and lower among children whose mothers reported respiratory tract, urinary tract, gastrointestinal, and other infections before or during pregnancy. Therefore, in our Latino population, the influence of direct exposure to infections during early life on AL development did not support the Greaves’ hypothesis. However, Greaves’ hypothesis was supported by the association of AL with children’s indirect exposure to infections, as could occur through the mother’s infections during or before pregnancy, especially for respiratory tract infections.

The majority of studies in non-Latino populations indicate that exposure to common infections in early life is associated with reduced AL risk [14,15,16,17,18,22,27]; however, the few studies in Latino populations have produced conflicting results [19,22,25]. While Ma et al. (2005) reported no association of infections with AL in Latino children residing in California, USA [22], Urayama et al. (2011) reported a reduced AL risk (OR, 0.40; 95% CI, 0.18–0.91) in children with ear infections before 6 months of age [25]. In a recent study of children in Costa Rica, Figueroa et al. (2020) reported a reduced risk of ALL when children were in contact with any pet or farm animal in early life (OR, 0.44; 95% CI, 0.31–0.62 and OR, 0.66; 95% CI, 0.49–0.90, respectively), and increased risk when children experienced a fever for longer than one week, which is a putative proxy of severe infection (OR, 2.44; 95% CI, 1.61–3.70) [19]. Similarly, in our population of Mexican children, we found that exposure to upper respiratory tract infections in early life was associated with increased risk of AL (OR, 2.76; 95% CI, 1.48–5.15), especially ALL (OR, 3.14; 95% CI, 1.67–5.89) and Pre-B (OR, 3.11; 95% CI, 1.63–5.96).

A growing body of evidence suggests that maternal inflammation and/or infection are perceived by fetal hematopoietic stem cells, thereby shaping the immune system during the neonatal period and beyond [28]. The majority of relevant studies in non-Latino populations have reported that maternal infection in pregnancy is positively associated with childhood leukemia. A meta-analysis including 32 articles describing 20 studies (ALL, *n* = 15; childhood leukemia, *n* = 14), reported that most studies showed a positive association between infection variables and ALL or childhood leukemia. Among specific types of infection, influenza during pregnancy was associated with a higher risk of ALL (pooled OR, 3.64; 95% CI, 1.34–9.90) and childhood leukemia (pooled OR, 1.77; 95% CI, 1.01–3.11). Higher childhood leukemia risk was also associated with varicella (pooled OR, 10.19; 95% CI, 1.98–52.39) and rubella (pooled OR, 2.79; 95% CI, 1.16–6.71) [29]. A later prospective study analyzed six population-based birth cohorts in Australia, Denmark, Israel, Norway, the UK, and the USA (recruitment in the 1950s–2000s, 167 acute leukemia cases), and reported that maternal urinary tract infection was associated with an increased risk of any leukemia (adjusted hazard ratio [HR], 1.68; 95% CI, 1.10–2.58) and of ALL (HR, 1.49; 95% CI, 0.87–2.56) and AML (HR, 2.70; 95% CI, 0.93–7.86), and that respiratory tract infection was associated with increased risk of any leukemia (HR, 1.57; 95% CI, 1.06–2.34), including subtype ALL (HR, 1.43; 95% CI, 0.94–2.19) and AML (HR, 2.37; 95% CI, 1.10–5.12) [30]. A recent study included 1215 children with AL from Denmark and reported an increased risk of leukemia among children whose mothers had infections during pregnancy (HR, 1.35; 95% CI, 1.04–1.77), as well as an increased childhood leukemia risk associated with maternal genital (HR, 2.42; 95% CI, 1.50–3.92) and urinary tract infections (HR, 1.65; 95% CI, 1.15–2.36), but not maternal respiratory tract, digestive, or other infections [31]. To our knowledge, our present study is the first to analyze the relationship between maternal infections during pregnancy and childhood leukemia in a Latino population. We found that maternal infections during pregnancy were associated with a reduced risk of AL (OR, 0.61; 95% CI, 0.52–0.72), including ALL (OR, 0.59; 95% CI, 0.50–0.70) and Pre-B (OR, 0.58; 95% CI, 0.49–0.69). These results contrast with the findings of a majority of studies among non-Latinos [29,30,31]. Notably, maternal respiratory and urinary tract infections were the types of infections associated with reduced AL risk in our study population. Similarly, maternal infections before pregnancy were also associated with a reduced risk of AL (OR, 0.55; 95% CI, 0.47–0.64), including ALL (OR, 0.56; 95% CI, 0.48–0.65), Pre-B (OR, 0.52; 95% CI, 0.44–0.62), and AML (OR, 0.50; 95% CI, 0.37–0.68). A stratified analysis revealed that respiratory tract infections were the type of infection that served as a protector factor against all kinds of leukemias. Gastrointestinal infections and other infections were also identified as protective factors but were not associated with AML.

According to an extensive review by Apostol et al. (2020), the maternal immune response to infection and inflammation may be perceived by the fetus at the fetal–maternal interface through the following different mechanisms: (1) direct exposure to maternal antigen via antibody–antigen complexes mediated by the neonatal Fc receptor; (2) receptors on the maternal side responding to pathogen-associated molecular patterns produced by pathogens, and maternal cytokines that signal to the fetal side via Toll-like receptors and specific cytokine receptors; (3) cytokines passing across the fetal-maternal interface and directly interacting with receptors on the fetal side, which may evoke a different cellular response on the fetal side; and (4) vertical transmission of infection from mother to fetus, causing immune cells to directly perceive and respond to the infection [28]. These mechanisms could be related to the association between maternal infections during pregnancy and the risk of AL development, but it is difficult to connect them to the findings regarding maternal infections before pregnancy. However, it has also been reported that early postnatal exposure to infection influences the risk of AL and that these effects extend into the uterus [20,32]. Based on this knowledge together with our present findings, we hypothesize that the window during which microbial exposure impacts the risk of AL development is not limited to early childhood infections, but could be extended to the zygote period. Further studies are needed to establish an association between maternal infections before pregnancy and leukemogenesis.

The paradox that infections are associated with AL sometimes as protective factors and sometimes as risk factors may have to do with several factors. Notably, some infectious agents, such as cytomegalovirus, may be more frequent in the Hispanic population and this may increase the risk of ALL in this population [33,34]. Another factor that influences developing countries and Latin countries is the indiscriminate use of antibiotics which induces important changes in the microbiome, which could increase or decrease the risk of ALL [33,34,35]. Moreover, the presence of a gene rearrangement during pregnancy may influence whether early exposure to some infectious agent will increase or decrease the risk of developing childhood ALL [33,34].

One of the main strengths of this study is that MIGICCL is a member of the Childhood Cancer & Leukemia International Consortium (CLIC), which has allowed the use of criteria very similar to those used in CLIC for most of the analyzed variables [36]. Additionally, the participation rate was over 90% for cases, and over 80% for controls, as previously described [37]. This decreases the chances of selection bias. Notably, we have lower sampling power to identify associations in cases of AML; however, the present work still includes a significant number of cases of this AL subtype. Another limitation of our study is the use of a broad categorization of infection. Thus, there remains a need for further studies among specific types of infections or infectious agents. One aspect to consider is that there may be a recall bias due to the fact that several years passed between the occurrences of infections and the time that children developed leukemia or were included as controls. However, if there is a bias, it should be a non-differential bias, since there is no evidence that parents of cases tend to remember infections more or less accurately than parents of controls. With respect to confounding biases, we were unable to analyze the microbiome at birth, which is a potentially important factor that could not be identified due to the retrospective nature of our study.

## 5. Conclusions

The present study in a Latino population revealed that upper respiratory tract infections during a child’s first year of life were associated with AL development in children of Mexico City. Moreover, the mothers’ exposure to respiratory tract infections before or during pregnancy was shown to be an important preventative factor against AL in our population. These results add to the growing evidence suggesting leukemia etiology differences between Latino and non-Latino populations. Additionally, our findings indicate a probable extension of the window effect during which infection incidence can influence the risk of AL development, but further studies are needed.

## Figures and Tables

**Table 1:** Sociodemographic characteristics and descriptive analysis of cases and controls residing in Mexico City (2002–2016).

Variables		AL			ALL			Pre-B ALL			AML		Controls	*P* (ꭓ^2^ Test)
	*n* = 1455	OR (95% CI)	*p*	*n* = 1253	OR (95% CI)	*p*	*n* = 1019	OR (95% CI)	*p*	*n* = 202	OR (95% CI)	*p*	*n* = 1455	
Sex														0.028
Female	677 (46.5%)	1	REF	585 (46.7%)	1	REF	495 (48.6%)	1	REF	92 (45.5%)	1	REF	618 (42.5%)	
Male	**778 (53.5%)**	**0.85 (0.73–0.98)**	**0.028**	**668 (53.3%)**	**0.84 (0.72–0.98)**	**0.028**	**524 (51.4%)**	**0.78 (0.66–0.92)**	**0.003**	110 (54.5%)	0.88 (0.66–1.19)	0.409	837 (57.5%)	
Age at diagnosis														<0.001
<5 years	568 (39%)	1	REF	514 (41%)	1	REF	427 (41.9%)	1	REF	54 (26.7%)	1	REF	644 (44.3%)	
5–15 years	**777 (53.4%)**	**1.16 (1.00–1.35)**	**0.053**	648 (51.7%)	1.07 (0.92–1.25)	0.398	514 (50.4%)	1.02 (0.87–1.21)	0.803	**129 (63.9%)**	**2.03 (1.45–2.83)**	**0.000**	759 (52.2%)	
>15 years	**110 (7.6%)**	**2.40 (1.69–3.40)**	**0.000**	**91 (7.3%)**	**2.19 (1.53–3.14)**	**0.000**	**78 (7.7%)**	**2.26 (1.56–3.28)**	**0.000**	**19 (9.4%)**	**4.36 (2.41–7.90)**	**0.000**	52 (3.6%)	
Birth weight														0.016
<3500 g	1027 (70.6%)	1	REF	890 (71%)	1	REF	714 (70.1%)	1	REF	137 (67.8%)	1	REF	1085 (74.6%)	
≥3500 g	**428 (29.4%)**	**1.22 (1.04–1.44)**	**0.016**	**363 (29%)**	**1.20 (1.00–1.42)**	**0.039**	**305 (29.9%)**	**1.25 (1.05–1.50)**	**0.013**	**65 (32.2%)**	**1.39 (1.01–1.91)**	**0.041**	370 (25.4%)	
First born														0.003
No	864 (59.4%)	1	REF	746 (59.5%)	1	REF	590 (57.9%)	1	REF	118 (58.4%)	1	REF	785 (54%)	
Yes	**591 (40.6%)**	**0.81 (0.69–0.93)**	**0.003**	**507 (40.5%)**	**0.80 (0.68–0.93)**	**0.003**	**429 (41.1%)**	**0.85 (0.73–1.00)**	**0.520**	84 (41.6%)	0.83 (0.62–1.12)	0.233	670 (46%)	
Family history of cancer														0.001
No	820 (56.4%)	1	REF	705 (56.3%)	1	REF	579 (56.8%)	1	REF	115 (56.9%)	1	REF	908 (62.4%)	
Yes	**635 (43.6%)**	**1.30 (1.11–1.50)**	**0.001**	**548 (43.7%)**	**1.29 (1.11–1.51)**	**0.001**	**440 (43.2%)**	**1.26 (1.07–1.49)**	**0.005**	87 (43.1%)	1.26 (0.93–1.70)	0.134	547 (37.6%)	
Low standard of living														0.850
No	597 (41%)	1	REF	509 (40.6%)	1	REF	431 (42.3%)	1	REF	88 (43.6%)	1	REF	592 (40.7%)	
Yes	858 (59%)	0.99 (0.85–1.14)	0.850	744 (59.4%)	1.00 (0.86–1.17)	0.970	588 (57.7%)	0.94 (0.80–1.10)	0.424	114 (56.4%)	0.89 (0.66–1.20)	0.440	863 (59.3%)	
Mother’s age at child’s birth														0.818
<35 years	1326 (91.3%)	1	REF	1138 (91%)	1	REF	919 (90.4%)	1	REF	188 (93.5%)	1	REF	1320 (90.8%)	
≥35 years	126 (8.7%)	0.94 (0.73–1.22)	0.653	113 (9%)	0.99 (0.76–1.28)	0.913	98 (9.6%)	1.06 (0.81–1.39)	0.685	13 (6.5%)	0.69 (0.38–1.24)	0.208	133 (9.2%)	
Father’s age at child’s birth														0.174
<35 years	1180 (81.4%)	1	REF	1015 (81.3%)	1	REF	828 (81.6%)	1	REF	165 (81.7%)	1	REF	1217 (83.8%)	
≥35 years	270 (18.6%)	1.19 (0.98–1.44)	0.083	233 (18.7%)	1.19 (0.97–1.45)	0.089	187 (18.4%)	1.17 (0.95–1.45)	0.146	37 (18.3%)	1.16 (0.79–1.70)	0.444	235 (16.2%)	
Mother’s education years														0.171
9.1–12.9 years	433 (29.8%)	1	REF	382 (30.5%)	1	REF	320 (31.4%)	1	REF	51 (25.2%)	1	REF	480 (33%)	
≥13 years	209 (14.4%)	1.15 (0.91–1.46)	0.232	181 (14.4%)	1.13 (0.89–1.44)	0.316	148 (14.5%)	1.10 (0.86–1.43)	0.445	28 (13.9%)	1.31 (0.80–2.14)	0.278	201 (13.8%)	
≤9 years	813 (55.9%)	1.16 (0.99–1.37)	0.067	690 (55.1%)	1.12 (0.95–1.33)	0.188	551 (54.1%)	1.07 (0.89–1.30)	0.472	**123 (60.9%)**	**1.50 (1.06–2.11)**	**0.022**	774 (53.2%)	
Father education’s years														0.012
9.1–12.9 years	388 (26.7%)	1	REF	330 (26.3)	1	REF	275 (27%)	1	REF	58 (28.7%)	1	REF	459 (31.5%)	
≥13 years	**234 (16.1%)**	**1.34 (1.06–1.68)**	**0.014**	**203 (16.2%)**	**1.36 (1.07–1.73)**	**0.011**	**172 (16.9%)**	**1.39 (1.08–1.78)**	**0.011**	31 (15.3%)	1.19 (0.74–1.89)	0.475	207 (14.2%)	
≤9 years	**833 (57.3%)**	**1.25 (1.06–1.48)**	**0.009**	**720 (57.5%)**	**1.27 (1.07–1.51)**	**0.007**	**572 (56.1%)**	**1.21 (1.01–1.56)**	**0.042**	113 (55.9%)	1.13 (0.81–1.59)	0.466	789 (54.2%)	
Breastfeeding														<0.001
No	125 (8.6%)	1	REF	101 (8.1%)	1	REF	80 (7.9%)	1	REF	24 (11.9%)	1	REF	183 (12.6%)	
Yes	**1330 (91.4%)**	**1.53 (1.20–1.95)**	**0.000**	**1152 (91.9%)**	**1.64 (1.27–2.12)**	**0**	**939 (92.1%)**	**1.69 (1.28–2.23)**	**0**	178 (88.1%)	1.07 (0.68–1.68)	0.779	1272 (87.4%)	
Duration														
0–3.9 months	410 (28.3%)	1	REF	354 (28.4%)	1	REF	294 (29.1%)	1	REF	56 (27.7%)	1	REF	488 (33.7%)	
4–6.9 months	**281 (19.4%)**	**1.38 (1.11–1.72)**	**0.003**	**246 (19.7%)**	**1.40 (1.12–1.75)**	**0.003**	**200 (19.8%)**	**1.37 (1.08–1.74)**	**0.009**	35 (17.3%)	1.26 (0.80–1.98)	0.313	242 (16.7%)	
7–12.9 months	**474 (32.7%)**	**1.21 (1.01–1.45)**	**0.043**	**408 (32.7%)**	**1.20 (1.00–1.46)**	**0.056**	325 (32.1%)	1.16 (0.94–1.41)	0.163	66 (32.7%)	1.23 (0.84–1.80)	0.280	467 (32.3%)	
≥13 months	**283 (19.5%)**	**1.35 (1.09–1.68)**	**0.006**	**238 (19.1%)**	**1.32 (1.05** **–** **1.65)**	**0.016**	**193 (19.1%)**	**1.29 (1.02–1.63)**	**0.037**	**45 (22.3%)**	**1.58 (1.03–2.40)**	**0.034**	249 (17.2%)	
Infection during child’s first year of life														0.295
No	1367 (94%)	1	REF	1169 (93.3%)	1	REF	952 (93.4%)	1	REF	198 (98%)	1	REF	1380 (94.8%)	
Yes	88 (6%)	1.18 (0.86–1.62)	0.295	84 (6.7%)	1.32 (0.96–1.82)	0.087	67 (6.6%)	1.30 (0.92–1.82)	0.135	4 (2%)	0.37 (0.13–1.03)	0.047	75 (5.2%)	
Type of infection														
None	1367 (94%)	1	REF	1169 (93.3%)	1	REF	952 (93.4%)	1	REF	198 (98%)	1	REF	1380 (94.8%)	
Upper respiratory tract infections	**37 (2.5%)**	**2.67 (1.44–4.96)**	**0.002**	**36 (2.9%)**	**3.04 (1.63–5.66)**	**0.000**	**30 (2.9%)**	**3.11 (1.64–5.89)**	**0.001**	1 (0.5%)	0.50 (0.07–3.80)	0.502	14 (1%)	
Lower respiratory tract infections	12 (0.8%)	0.93 (0.42–2.05)	0.861	11 (0.9%)	1.00 (0.45–2.24)	0.998	10 (1%)	1.12 (0.49–2.55)	0.797	1 (0.5%)	0.54 (0.07–4.12)	0.549	13 (0.9%)	
Gastrointestinal infections	20 (1.4%)	1.06 (0.57–2.00)	0.850	19 (1.5%)	1.18 (0.62–2.24)	0.612	13 (1.3%)	0.99 (0.49–2.02)	0.982	1 (0.5%)	0.37 (0.05–2.76)	0.330	19 (1.3%)	
Others	19 (1.3%)	0.66 (0.37–1.19)	0.165	18 (1.4%)	0.73 (0.41–1.33)	0.304	14 (1.4%)	0.70 (0.37–1.33)	0.277	1 (0.5%)	0.24 (0.03–1.77)	0.162	29 (2%)	
Hospitalization by infection during child’s first year of life														0.159
No	1433 (98.6%)	1	REF	1232 (98.4%)	1	REF	1002 (98.4%)	1	REF	201 (99.5%)	1	REF	1426 (98.4%)	
Yes	21 (1.4%)	0.91 (0.50–1.65)	0.753	20 (1.6%)	1.01 (0.55–1.84)	0.983	16 (1.6%)	0.99 (0.52–1.88)	0.976	1 (0.5%)	0.31 (0.41–2.30)	0.224	23 (1.6%)	
Type of infection														
None	1434 (98.6%)	1	REF	1233 (98.4%)	1	REF	1003 (98.4%)	1	REF	201 (99.5%)	1	REF	1432 (98.4%)	
Upper respiratory tract infections	5 (0.3%)	0.83 (0.25–2.73)	0.762	5 (0.4%)	0.97 (0.30–3.18)	0.957	3 (0.3%)	0.71 (0.18–2.86)	0.634	0 (0%)	Undefined	0.999	6 (0.4%)	
Lower respiratory tract infections	7 (0.5%)	1.75 (0.51–5.98)	0.374	6 (0.5%)	1.74 (0.50–6.19)	0.391	6 (0.6%)	2.14 (0.60–7.61)	0.239	1 (0.5%)	1.78 (0.20–16.01)	0.606	4 (0.3%)	
Gastrointestinal infections	6 (0.4%)	1.20 (0.37–3.94)	0.766	6 (0.5%)	1.39 (0.42–4.58)	0.584	4 (0.4%)	1.14 (0.31–4.26)	0.843	0 (0%)	Undefined	0.999	5 (0.3%)	
Others	3 (0.2%)	0.37 (0.10–1.41)	0.147	3 (0.2%)	0.44 (0.12–1.65)	0.220	3 (0.3%)	0.54 (0.14–2.02)	0.357	0 (0%)	Undefined	0.999	8 (0.5%)	
Mother’s infections before pregnancy														<0.001
No	707 (48.6%)	1	REF	601 (48%)	1	REF	501 (49.2%)	1	REF	106 (52.5%)	1	REF	494 (34%)	
Yes	**748 (51.4%)**	**0.54 (0.47–0.63)**	**0.000**	**652 (52%)**	**0.56 (0.48–0.65)**	**0.000**	**518 (50.8%)**	**0.53 (0.45–0.63)**	**0.000**	**96 (47.5%)**	**0.47 (0.35–0.63)**	**0.000**	961 (66%)	
Type of infections														
None	702 (48.2%)	1	REF	594 (47.4%)	1	REF	497 (48.8%)	1	REF	108 (53.5%)	1	REF	478 (32.9%)	
Respiratory tract infections	**691 (47.5%)**	**0.53 (0.46–0.62)**	**0.000**	**607 (48.4%)**	**0.55 (0.47–0.65)**	**0.000**	**482 (47.3%)**	**0.52 (0.44–0.62)**	**0.000**	**84 (41.6%)**	**0.42 (0.31–0.57)**	**0.000**	886 (60.9%)	
Gastrointestinal infections	**30 (2.1%)**	**0.39 (0.24–0.61)**	**0.000**	**23 (1.8%)**	**0.35 (0.21–0.58)**	**0.000**	**18 (1.8%)**	**0.33 (0.19–0.57)**	**0.000**	7 (3.5%)	0.59 (0.26–1.32)	0.197	53 (3.6%)	
Urinary tract infections	17 (1.2%)	0.83 (0.40–1.70)	0.603	15 (1.2%)	0.86 (0.41–1.80)	0.694	11 (1.1%)	0.76 (0.34–1.68)	0.492	2 (1%)	0.63 (0.14–2.82)	0.548	14 (1%)	
Vaginal infections	8 (0.5%)	1.36 (0.41–4.55)	0.616	7 (0.6%)	1.41 (0.41–4.84)	0.59	7 (0.7%)	1.68 (0.49–5.79)	0.409	1 (0.5%)	1.11 (0.12–9.99)	0.928	4 (0.3%)	
Others	**7 (0.5%)**	**0.24 (0.10–0.57)**	**0.001**	**7 (0.6%)**	**0.28 (0.12–0.67)**	**0.004**	**4 (0.4%)**	**0.19 (0.07–0.57)**	**0.003**	0 (0%)	Undefined	0.998	20 (1.4%)	
Mother’s infections during pregnancy														<0.001
No	616 (42.4%)	1	REF	537 (42.9%)	1	REF	440 (43.2%)	1	REF	79 (39.1%)	1	REF	443 (30.4%)	
Yes	**838 (57.6%)**	**0.60 (0.51–0.69)**	**0.000**	**715 (57.1%)**	**0.58 (0.50–0.69)**	**0.000**	**579 (56.8%)**	**0.58 (0.49–0.68)**	**0.000**	**123 (60.9%)**	**0.68 (0.50–0.92)**	**0.013**	1012 (69.6%)	
Type of infections														
None	609 (41.9%)	1	REF	530 (42.3%)	1	REF	433 (42.5%)	1	REF	79 (39.1%)	1	REF	438 (30.1%)	
Respiratory tract infections	**525 (36.1%)**	**0.58 (0.49–0.69)**	**0.000**	**447 (35.7%)**	**0.57 (0.48–0.68)**	**0.000**	**360 (35.3%)**	**0.56 (0.47–0.68)**	**0.000**	**78 (38.6%)**	**0.67 (0.48–0.93)**	**0.018**	648 (44.5%)	
Gastrointestinal infections	60 (4.1%)	0.70 (0.48–1.01)	0.059	56 (4.5%)	0.75 (0.51–1.10)	0.134	42 (4.1%)	0.69 (0.45–1.04)	0.073	4 (2%)	0.36 (0.13–1.01)	0.052	62 (4.3%)	
Urinary tract infections	**232 (15.9%)**	**0.59 (0.47–0.72)**	**0.000**	**194 (15.5%)**	**0.56 (0.45–0.70)**	**0.000**	**161 (15.8%)**	**0.57 (0.45–0.72)**	**0.000**	38 (18.8%)	0.74 (0.49–1.12)	0.150	285 (19.6%)	
Vaginal infections	23 (1.6%)	1.03 (0.54–1.98)	0.92	20 (1.6%)	1.03 (0.53–2.02)	0.924	17 (1.7%)	1.08 (0.54–2.16)	0.84	3 (1.5%)	1.04 (0.30–3.65)	0.952	16 (1.1%)	
Others	6 (0.4%)	0.72 (0.23–2.25)	0.57	6 (0.5%)	0.83 (0.27–2.58)	0.743	6 (0.6%)	1.01 (0.32–3.16)	0.984	0 (0%)	Undefined	0.999	6 (0.4%)	

AL: acute leukemia; ALL: acute lymphoblastic leukemia; ALL Pre-B: Precursor B-cell acute lymphoblastic leukemia; AML: acute myeloblastic leukemia; *n*: sample number; CI: confidence intervals; OR: odds ratio; REF: reference value; *p*: *p*-value. Bolded values indicate odds ratios for variables with an identified association.

**Table 2:** Logistic regression model for infections during child’s first year of life.

	AL			ALL			ALL PreB			AML		
	Children	OR (95% CI)	*p*	Children	OR (95% CI)	*p*	Children	OR (95% CI)	*p*	Children	OR (95% CI)	*p*
Infections during child’s first year of life												
No	2742	1	REF	2545	1	REF	2328	1	REF	1575	1	REF
Yes	163	1.22 (0.89–1.69)	0.221	159	1.36 (0.98–1.89)	0.065	142	1.30 (0.92–1.84)	0.139	79	0.42 (0.15–1.19)	0.102
Type of infection												
None	2742	1	REF	2545	1	REF	2328	1	REF	1575	1	REF
Upper respiratory tract infections	**51**	**2.76 (1.48–5.15)**	**0.001**	**50**	**3.14 (1.67–5.89)**	**<0.001**	**44**	**3.11 (1.63–5.96)**	**0.001**	15	0.58 (0.07–4.49)	0.601
Lower respiratory tract infections	25	0.91 (0.41–2.02)	0.817	24	0.98 (0.43–2.21)	0.952	23	1.09 (0.47–2.52)	0.847	14	0.51 (0.07–3.99)	0.522
Gastrointestinal infections	39	1.12 (0.59–2.13)	0.724	38	1.25 (0.65–2.38)	0.509	32	1.04 (0.51–2.13)	0.919	20	0.40 (0.05–3.06)	0.376
Others	48	0.69 (0.38–1.24)	0.215	47	0.75 (0.41–1.38)	0.359	43	0.69 (0.36–1.33)	0.266	30	0.31 (0.04–2.32)	0.254
Hospitalization by infection during child’s first year of life												
No	2854	1	REF	2654	1	REF	2424	1	REF	1624	1	REF
Yes	44	0.92 (0.50–1.68)	0.778	43	1.01 (0.55–1.86)	0.983	39	0.99 (0.52–1.91)	0.98	24	0.38 (0.50–2.85)	0.345
Type of infection												
None	2861	1	REF	2661	1	REF	2431	1	REF	1630	1	REF
Upper respiratory tract infections	11	0.75 (0.23–2.50)	0.641	11	0.87 (0.26–2.87)	0.812	9	0.61 (0.15–2.49)	0.488	6	5.48 × 10^−9^ (0.00–0.00)	0.999
Lower respiratory tract infections	11	1.85 (0.53–6.42)	0.334	10	1.80 (0.50–6.49)	0.371	10	2.16 (0.60–7.83)	0.241	5	2.39 (0.26–22.06)	0.443
Gastrointestinal infections	11	1.15 (0.35–3.81)	0.820	11	1.36 (0.41–4.52)	0.613	9	1.19 (0.32–4.50)	0.796	5	4.41 × 10^−9^ (0.00–0.00)	0.999
Others	11	0.42 (0.11–1.62)	0.209	11	0.48 (0.13–1.85)	0.289	11	0.59 (0.16–2.26)	0.444	8	6.06 × 10^−9^ (0.00–0.00)	0.999

AL: acute leukemia; ALL: acute lymphoblastic leukemia; ALL Pre-B: Precursor B-cell acute lymphoblastic leukemia; AML: acute myeloblastic leukemia; CI: confidence intervals; OR: odds ratio; REF: reference value; *p*: *p*-value. Bolded values indicate odds ratios for variables with an identified association.

**Table 3:** Logistic regression model for mother’s infections before and during pregnancy.

	AL			ALL			ALL Pre-B			AML		
Variable	Children	OR (95% CI)	*p*	Children	OR (95% CI)	*p*	Children	OR (95% CI)	*p*	Children	OR (95% CI)	*p*
Mother’s infections before pregnancy												
No	1197	1	REF	1092	1	REF	992	1	REF	597	1	REF
Yes	**1708**	**0.55 (0.47–0.64)**	**<0.001**	**1612**	**0.56 (0.48–0.65)**	**<0.001**	**1478**	**0.52 (0.44–0.62)**	**<0.001**	**1057**	**0.50 (0.37–0.68)**	**<0.001**
Type of infection												
None	1176	1	REF	1069	1	REF	972	1	REF	583	1	REF
Respiratory tract infections	**1577**	**0.54 (0.46–0.63)**	**<0.001**	**1493**	**0.55 (0.47–0.65)**	**5 × 10^−13^**	**1368**	**0.51 (0.43–0.61)**	**3 × 10^−14^**	**970**	**0.45 (0.33–0.62)**	**6 × 10^−7^**
Gastrointestinal infections	**82**	**0.38 (0.24–0.61)**	**0.000**	**75**	**0.34 (0.20–0.57)**	**0.000**	**70**	**0.30 (0.17–0.54)**	**0.000**	60	0.61 (0.26–1.38)	0.234
Urinary tract infections	31	0.92 (0.44–1.91)	0.822	29	0.95 (0.45–2.01)	0.889	25	0.79 (0.35–1.78)	0.566	16	0.83 (0.18–3.85)	0.81
Vaginal infections	12	1.43 (0.43–4.79)	0.565	11	1.44 (0.42–4.97)	0.566	11	1.71 (0.49–5.92)	0.397	5	1.26 (0.14–11.74)	0.841
Others	**27**	**0.26 (0.11–0.62)**	**0.002**	**27**	**0.30 (0.12–0.72)**	**0.007**	**24**	**0.20 (0.07–0.60)**	**0.004**	20	3.32 × 10^−9^ (0.00–0.00)	0.998
Mother’s infections during pregnancy												
No	1055	1	REF	977	1	REF	880	1	REF	520	1	REF
Yes	**1849**	**0.61 (0.52–0.72)**	**<0.001**	**1726**	**0.59 (0.50–0.70)**	**<0.001**	**1590**	**0.58 (0.49–0.69)**	**<0.001**	1134	0.80 (0.58–1.10)	0.167
Type of infection												
None	1043	1	REF	965	1	REF	868	1	REF	515	1	REF
Respiratory tract infections	**1173**	**0.60 (0.50–0.71)**	**<0.001**	**1095**	**0.57 (0.48–0.69)**	**<0.001**	**1008**	**0.56 (0.46–0.68)**	**<0.001**	726	0.79 (0.56–1.12)	0.19
Gastrointestinal infections	121	0.71 (0.49–1.05)	0.085	117	0.77 (0.52–1.14)	0.187	103	0.72 (0.47–1.09)	0.122	65	0.39 (0.14–1.12)	0.08
Urinary tract infections	**517**	**0.61 (0.49–0.76)**	**<0.001**	**479**	**0.58 (0.46–0.72)**	**<0.001**	**446**	**0.58 (0.46–0.74)**	**<0.001**	323	0.86 (0.56–1.31)	0.473
Vaginal infections	39	1.11 (0.57–2.14)	0.767	36	1.08 (0.55–2.12)	0.834	33	1.13 (0.56–2.29)	0.742	19	1.38 (0.38–4.97)	0.624
Others	12	0.67 (0.21–2.15)	0.499	12	0.78 (0.24–2.49)	0.669	12	0.94 (0.29–3.02)	0.92	6	3.98 × 10^−9^ (0.00–0.00)	0.999

AL: acute leukemia; ALL: acute lymphoblastic leukemia; ALL Pre-B: Precursor B-cell acute lymphoblastic leukemia; AML: acute myeloblastic leukemia; CI: confidence intervals; OR: odds ratio; REF: reference value; *p*: *p*-value. Bolded values indicate odds ratios for variables with an identified association.

## Data Availability

The datasets generated and analyzed during the current study are not publicly available but are available from the authors on reasonable request.
